# Molecular characteristics and antimicrobial susceptibility of carbapenem-resistant *Klebsiella pneumoniae* in a multicenter study in Ningbo, China

**DOI:** 10.3389/fmicb.2025.1628592

**Published:** 2025-07-21

**Authors:** Hongfei Shi, Yanye Tu, Hong Li, Hui Gao, Feng Wang, Wei Zhang, Min Jiang, Qian Sun, Zheng Bao, Xiangwei Yang, Yanzi Chang

**Affiliations:** ^1^Department of Clinical Laboratory, The Affiliated Li Huili Hospital of Ningbo University, Ningbo, China; ^2^Department of Clinical Laboratory, The Affiliated Ningbo Women and Children’s Hospital of Ningbo University, Ningbo, China; ^3^Department of Clinical Laboratory, Xiangshan Red Cross Taiwan Compatriot Hospital Medical and Health Group, Ningbo, China; ^4^Department of Clinical Laboratory, Second Hospital of Ninghai County, Ningbo, China

**Keywords:** *Klebsiella pneumoniae*, carbapenem resistance, resistance genes, virulence genes, plasmids introduction

## Abstract

**Objective:**

To analyze the molecular epidemiology and antimicrobial resistance profiles of carbapenem-resistant *Klebsiella pneumoniae* (CR-KP) isolates in Ningbo, with the aim of providing a theoretical basis for hospital infection control strategies and the implementation of precise clinical diagnosis and treatment protocols.

**Methods:**

During the period from April 30, 2023 to June 30, 2024, clinical isolates of *Klebsiella pneumoniae* were collected from multiple centers in Ningbo, including The Affiliated Li Huili Hospital (Yinzhou District, Ningbo), Xiangshan Red Cross Taiwan Compatriot Hospital Medical and Health Group (Xiangshan, Ningbo), and the Second Hospital of Ninghai County (Ninghai, Ningbo). A total of 81 CR-KP strains were identified using the broth dilution method for carbapenem resistance screening. These isolates were submitted to Beijing Novo gene Co., Ltd. for sequencing analysis. The sequencing data were analyzed using online tools (https://bigsdb.pasteur.fr/ and http://genepi.food.dtu.dk/resfinder) to obtain information on multilocus sequence typing (MLST), capsular serotype (KL type), virulence genes, and resistance genes. Phylogenetic relationships were constructed using SNP software. For plasmid characterization, the PlasmidFinder online tool (https://cge.food.dtu.dk/services/PlasmidFinder/) was utilized to identify plasmid replicon genes and perform Inc. typing analysis. Furthermore, to conduct a comprehensive collinearity analysis of the *bla*_KPC-2_ resistance plasmid gene, gene cluster maps were constructed using Bakta v1.11.0 and Clinker v0.0.28 software packages.

**Results:**

Among the 81 CR-KP isolates, MLST typing revealed that ST11 was the predominant sequence type, accounting for 66.67% (54/81), with KL64 being the dominant capsular type. Among the non-ST11 CR-KP isolates, the ST15 type accounted for 48.15% (13/27), with KL19 being the predominant capsular serotype. The carriage rate of virulence genes—including *rmpA2*, *fyuA*, and 10 other genes—was significantly higher in ST11 CR-KP compared to non-ST11 CR-KP (*p* < 0.05). Analysis of resistance genes revealed that ST11 CR-KP primarily carried *bla*_KPC-2_ (100%, 54/54), whereas the resistance gene profiles among non-ST11 CR-KP isolates were more diverse, including *bla_NDM_*, *bla*_IMP_, and *bla*_OXA_. Plasmid typing indicated that ST11 CR-KP predominantly harbored IncFII (98.15%, 53/54) and RepB (72.22%, 39/54) plasmid types. In contrast, non-ST11 CR-KP isolates exhibited a wider range of plasmid types, including IncX3 (33.33%, 9/27), RepB (25.93%, 7/27), IncFII (25.93%, 7/27), IncFIB (7.41%, 2/27), and both ColKP3 and Col440II (7.41%, 2/27). Antimicrobial susceptibility testing demonstrated high resistance rates to commonly used antibiotics in both ST11 and non-ST11 CR-KP isolates. ST11 CR-KP exhibited 100% resistance to six antibiotics, including ceftriaxone (CRO), cefotetan (CTT), and cefepime (FEP), and showed susceptibility only to gentamicin (GEN), aztreonam/avibactam (AZA), ceftazidime/avibactam (CZA), polymyxin B (POL), and tigecycline (TGC). Non-ST11 CR-KP showed a significantly higher resistance rate to gentamicin (GEN) and ceftazidime/avibactam (CZA) than ST11 CR-KP (*p* < 0.05), but lower resistance rates to cefotetan (74.07%), all of which were statistically significant (*p* < 0.05).

**Conclusion:**

In the Ningbo region, CR-KP is predominantly of the ST11-KL64 type, exhibiting both strong antimicrobial resistance and high virulence characteristics. Non-ST11 CR-KP isolates carry genetically diverse carbapenemase genes and mobile genetic elements (e.g., IncX3, ColKP3). ST11 CR-KP strains demonstrate significantly stronger resistance profiles compared to non-ST11 strains. Therefore, stringent control over the use of carbapenem antibiotics is essential, along with measures to prevent the spread of resistance plasmids and the continuous improvement of hospital infection control strategies.

## 1 Introduction

*Klebsiella pneumoniae* (KP), a common opportunistic pathogen, is widely colonized in the human gastrointestinal, respiratory, and genitourinary tracts. It is a major causative agent of various clinical infections, including pneumonia, urinary tract infections, and meningitis (Zhu et al., 2025). In recent years, with the widespread use of broad-spectrum antibiotics, carbapenem-resistant *Enterobacteriaceae* (CRE) have emerged as a major global public health threat. Due to their broad-spectrum resistance, high mortality rates, and limited treatment options, CRE have been classified by the World Health Organization as a “critical priority” group of pathogens (Zhai and Wu, 2024). According to data from the China Antimicrobial Surveillance Network (CHINET) (http://www.chinets.com) (Guo et al., 2024), the resistance rate of KP to imipenem surged from 3.0% in 2005 to 24.8% in 2023, representing an 8.26-fold increase. Apart from relatively low resistance rates to ceftazidime/avibactam (11%), polymyxin B (10.9%), and tigecycline (4.6%), carbapenem-resistant *Klebsiella pneumoniae* (CR-KP) exhibits resistance rates exceeding 50% to most other antimicrobial agents. Notably, resistance rates to ceftriaxone, cefepime, cefoperazone/sulbactam, and piperacillin/tazobactam exceed 90% (Guo et al., 2024). This alarming resistance profile renders frontline clinical treatment of CR-KP infections extremely challenging, with treatment failures in eradicating the pathogen resulting in mortality rates as high as 40–50% (Wu C. et al., 2022).

Since the first report of KPC-type carbapenemase in the United States in 1996, CR-KP strains producing KPC-type carbapenemase have disseminated widely across the globe (Wendt et al., 2010; Labarca et al., 2014). In 2005, France reported its first case of CR-KP harboring KPC-2, and in 2007, the first isolation of KPC-producing *K. pneumoniae* in China was documented by Li Lanjuan’s team (Wei et al., 2007). In recent years, the molecular epidemiological profile of CR-KP in China has shown that *bla*_KPC-2_ remains the predominant carbapenemase gene, with the ST11 clone being the most prevalent lineage (Zhang et al., 2017; Wang et al., 2018). Of particular concern is the rising clinical isolation rate of hypervirulent *Klebsiella pneumoniae* (hvKP) and the emergence of carbapenem-resistant hypervirulent *Klebsiella pneumoniae* (CR-hvKP), a class of “superbugs” exhibiting both hypervirulence and high-level antimicrobial resistance, which are rapidly spreading on a global scale (Wu Y. et al., 2022; Pu et al., 2023a; Behera et al., 2024). Recent studies have identified that CR-hvKP can harbor two or even three carbapenemase genes simultaneously, such as *bla_OXA- + KPC_*, or combinations like *bla_KPC + OXA+NDM_* (Genç et al., 2022; Li et al., 2023; Moussa et al., 2023). A 2024 study published in *Microbiological Research* revealed a plasmid fusion mechanism mediated by IncN-like plasmids, facilitating the horizontal transfer of virulence plasmids. This mechanism may drive the convergence of multidrug resistance and hypervirulence in *K. pneumoniae* (Xu et al., 2024).

Our previous investigations confirmed the emergence and dissemination of CR-hvKP strains, predominantly of the ST11-KL64 type, in the Ningbo region. These strains exhibit not only extensive antimicrobial resistance but also co-harbor multiple resistance and virulence genes, posing a risk for co-transfer of resistance-virulence plasmids (Jiang et al., 2024; Tu et al., 2024). Therefore, it is of significant clinical and public health importance to conduct an in-depth investigation into the molecular epidemiological characteristics and antimicrobial susceptibility of CR-KP across multiple centers in Ningbo. The objective of this study is to elucidate the molecular epidemiology and resistance profiles of CR-KP in Ningbo, thereby providing a theoretical foundation for rational antibiotic use, enhanced resistance surveillance, hospital infection prevention and control, and curbing the co-transmission of resistance and virulence plasmids.

## 2 Materials and methods

### 2.1 Materials

#### 2.1.1 Bacterial strain collection and selection

A total of 81 clinical isolates of CR-KP were collected from multiple medical centers in the Ningbo region from April 30, 2023 to June 30, 2024. These included 44 isolates from The Affiliated Li Huili Hospital (Yinzhou District, Ningbo), 21 isolates from Xiangshan Red Cross Taiwan Compatriot Hospital Medical And Health Group (Xiangshan, Ningbo), and 16 isolates from the Second Hospital of Ninghai County (Ninghai, Ningbo). Isolation and identification were performed using matrix-assisted laser desorption/ionization time-of-flight mass spectrometry (MALDI-TOF MS, EXS3600). Isolates identified as *Klebsiella pneumoniae* were included in the study. *In vitro* antimicrobial susceptibility testing was conducted using the broth dilution method. Isolates resistant to imipenem were further confirmed by the imipenem disk diffusion method. Interpretation of antimicrobial susceptibility results followed the Clinical and Laboratory Standards Institute (CLSI) guidelines (M100-Ed33) (Rai et al., 2023). Isolates were classified as carbapenem-resistant if the minimum inhibitory concentration (MIC) of imipenem was ≥4 μg/mL and the inhibition zone diameter in the Kirby-Bauer disk diffusion test (K-B method) was ≤19 mm. Specimens derived from a single patient were omitted during the analytical process. Procedural ethical compliance was confirmed by the Research Ethics Panel of The Affiliated Li Huili Hospital (Approval No. KY2022SL390-01).

#### 2.1.2 Instruments and reagents

The research utilized an EXS3600 microbial mass spectrometry detection instrument, procured through Zybio Inc., alongside the VITEK-2 Compact automated microbial identification system, obtained from bioMérieux (France); the imipenem antimicrobial susceptibility disks were purchased from Oxoid Ltd. (UK) (Batch No. 3768941); customized antimicrobial susceptibility cards for Gram-negative bacilli were obtained from Bio-Kont (Wenzhou, China) (Batch No. DZ1548); next-generation sequencing services were provided by Novogene Co., Ltd. (Beijing, China). The quality control strains *Escherichia coli* (ATCC 25922) and *Pseudomonas aeruginosa* (ATCC 27853) were preserved in the institutional biobank of microbial strains.

### 2.2 Methods

#### 2.2.1 Next-generation sequencing

Genomic DNA was extracted using the sodium dodecyl sulfate (SDS) method. Nucleic acid sample verification was conducted through agarose gel electrophoretic analysis, with subsequent molecular concentration determination performed using the Qubit fluorometric instrumentation. Following electrophoretic quality validation, genomic DNA specimens underwent systematic fragmentation to approximately 350 base pair lengths utilizing a Covaris ultrasonication instrument. The resulting DNA fragments were subjected to library preparation using the NEBNext^®^Ultra^™^ DNA Library Prep Kit for Illumina (NEB, United States), which included end repair, A-tailing, adapter ligation, purification, and PCR amplification steps to complete the construction of the sequencing library. After library construction was completed, preliminary quantification was performed using Qubit 2.0, and the library was diluted to a concentration of 2 ng/μL. The Agilent 2100 system was then used to evaluate the insert size of the library fragments. Once the insert size met expectations, the effective concentration of the library was accurately quantified using quantitative PCR (Q-PCR) to ensure library quality. Library preparations meeting quality specifications were aggregated based on their effective concentrations and anticipated data generation, subsequently undergoing paired-end 150 base pair sequencing via the Illumina NovaSeq platform. The method adheres to the fundamental principles of the classic (Maniatis et al., 1982; Sambrook and Russell, 2001) protocol while citing more modern and optimized approaches (Ashburner et al., 2000; Bairoch and Apweiler, 2000; Besemer et al., 2001; Simpson et al., 2009; Akhter et al., 2012; Bankevich et al., 2012; Galperin et al., 2015; Ge et al., 2016). The next-generation sequencing was carried out by Novogene Co., Ltd. (Beijing, China).

#### 2.2.2 Detection of capsular serotype and virulence genes

Sequencing data were submitted to the Pasteur Institute database (https://bigsdb.pasteur.fr/) to identify the capsular serotype (KL type) and virulence genes of the target *K. pneumoniae* strains. The virulence genes analyzed included capsule-associated regulatory genes *rmpA* and *rmpA2*; siderophore-related genes *fyuA, irp1, irp2, iucA, iucB, iucC, iucD, iutA*, and *ybtS*; and fimbriae-associated adhesion genes *mrkA, mrkB, mrkC, mrkD, allS*, and *allR*.

#### 2.2.3 Detection of antimicrobial resistance genes

Sequencing data were submitted to the ResFinder database (http://genepi.food.dtu.dk/resfinder) to identify antimicrobial resistance genes in the target strains. Analysis focused on carbapenemase resistance genes, including class A (*bla*_KPC_), class B (*bla_IPM_/bla*_NDM_*/bla*_VIM_), and class D (*bla*_OXA_); ESBL genes such as *bla*_TEM_, *bla*_SHV_, *bla*_CTX-M_; quinolone resistance genes including *gyrB, parC, parE*, and *qnrS*; aminoglycoside resistance genes *aph (3′)-Ia, aph (3′)-IIa, aac (3)-IId, aac (6′)-Ib-cr, armA*, and *aph (6)-Id;* and tetracycline resistance genes including *tet(A)*, *tmexD1* and *TOprJ1.*

#### 2.2.4 MLST homology analysis

Sequencing data were submitted to the Pasteur Institute database (https://bigsdb.pasteur.fr/) to obtain allele codes. These were compared against the database based on the allelic profiles of seven housekeeping genes (*gapA, infB, mdh, pgi, phoE, rpoB*, and *tonB*) to determine the multilocus sequence typing (MLST) type of the target strains. A phylogenetic tree was subsequently constructed using SNP software, and construct a minimum spanning tree based on the MLST allelic profile using Grape-Tree (Zhou et al., 2018).

#### 2.2.5 wgSNP-based phylogenetic analysis

Whole-genome single-nucleotide polymorphism (SNP) analysis of *Klebsiella pneumoniae* isolates was conducted using the Snippy v4.6.0 pipeline (https://github.com/tseemann/snippy). To account for potential phylogenetic distortions caused by homologous recombination, recombinant regions within the filtered alignment files were identified and masked using Gubbins v2.3.4 (Croucher et al., 2015). A maximum-likelihood phylogenetic tree was subsequently reconstructed from the core SNP alignment dataset using Fast-Tree 2 (Price et al., 2010). For optimal modeling of nucleotide substitutions, the General Time Reversible (GTR) evolutionary model (−gtr) was applied, with sequence data explicitly defined as nucleotides (−nt) to ensure accurate representation of strain-level evolutionary relationships.

#### 2.2.6 Comprehensive characterization and comparative analysis of plasmid genomes

PlasmidFinder (https://cge.food.dtu.dk/services/PlasmidFinder/) was utilized to identify plasmid replicon genes, in accordance with previously established methodologies (Rabiu et al., 2025; Yin et al., 2025). Following plasmid characterization, the assembled sequences were subjected to screening for the *bla*_KPC-2_ gene using ResFinder v4.1. To facilitate comprehensive genomic annotation of the relevant regions, Bakta v1.11.0 was employed for in-depth analysis. Additionally, Clinker v0.0.28 was used to generate comparative gene cluster maps, providing a visual representation of the genetic architecture surrounding the *bla*_KPC-2_ locus.

#### 2.2.7 Antimicrobial susceptibility testing

Antimicrobial susceptibility testing was systematically carried out using precision-engineered Gram-negative susceptibility screening panels (purchased from Bio-Kont, Wenzhou, China). Minimum inhibitory concentrations (MICs) of 12 antimicrobial agents were precisely evaluated against CR-KP strains utilizing the standardized broth microdilution methodology. The antimicrobial agents and their respective concentration ranges are listed in Table 1. MIC breakpoints were interpreted according to the Clinical and Laboratory Standards Institute (CLSI) M100-Ed33 guidelines, while tigecycline was evaluated based on the criteria provided by the U. S. Food and Drug Administration (FDA) (FDA, 2023). To characterize different resistance patterns, the isolates were classified as multi drug-resistant (MDR), extensively drug-resistant (XDR), and pan drug-resistant (PDR). MDR was defined as non-susceptibility to at least three different antimicrobial categories. XDR indicated susceptibility to only one or two antimicrobial categories, while PDR denoted non-susceptibility to all tested antimicrobial agents (Magiorakos et al., 2012).

**Table 1:** Antimicrobial susceptibility testing: antimicrobial class, concentration ranges, and breakpoints.

Antimicrobial class	Antimicrobials	Concentration ranges (μg/ml)	MIC breakpoints (μg/ml)		Reference standard
			Sensitivity (S)	Resistance (R)	
β-lactam	CTT^a^	0.125–256	≤16	≥64	CLSI
	CRO^a^	0.064–128	≤1	≥4	CLSI
	FEP^a^	0.064–128	≤2	≥16	CLSI
	TZP^a^	0.125/4–256/4	≤8/4	≥32/4	CLSI
	IPM^a^	0.125–256	≤1	≥4	CLSI
	AZA^a^	0.064/4–128/4	≤4/4	≥16/4	CLSI
	CZA^a^	0.25/4–256/4	≤8/4	≥32/4	CLSI
Aminoglycosides	GEN^a^	0.125–256	≤2	≥8	CLSI
Quinolone	LVX^a^	0.008–16	≤0.5	≥2	CLSI
	CIP^a^	0.008–16	≤0.25	≥1	CLSI
Polypeptide	POL^a^	0.064–64	-	≥4	CLSI
Tetracycline	TGC^b^	0.032–32	≤2	≥8	FDA

CTT, cefotetan; CRO, ceftriaxone; FEP, cefepime; TZP, piperacillin/tazobactam; IPM, imipenem; GEN, gentamicin; LVX, levofloxacin; CIP, ciprofloxacin; AZA, aztreonam/avibactam; CZA, ceftazidime/avibactam; POL, Polymyxin B; TGC, tigecycline.

a CLSI, Clinical and Laboratory Standards Institute.

b FDA, Food and Drug Administration.

#### 2.2.8 String test

Using a 1 μL inoculation loop, streak a single colony from an overnight culture in a vertical direction. Repeat this procedure three times to confirm the accuracy of the experiment. If a string ≥ 5 mm in length is formed when the colony is lifted, the result is considered positive.

#### 2.2.9 Statistical analysis

Employing Excel and SPSS version 29.0, the empirical data were systematically examined and statistically evaluated. Categorical variables were expressed as counts (n) or percentages (%), and comparisons were made using the χ^2^ test. Should the χ^2^ test’s fundamental assumptions fail to be met, researchers utilized Fisher’s exact test for statistical inference. Outcomes were interpreted as statistically substantive when the *P* < 0.05 demarcation point.

## 3 Results

### 3.1 Basic clinical data of isolates

**Figure 1:**
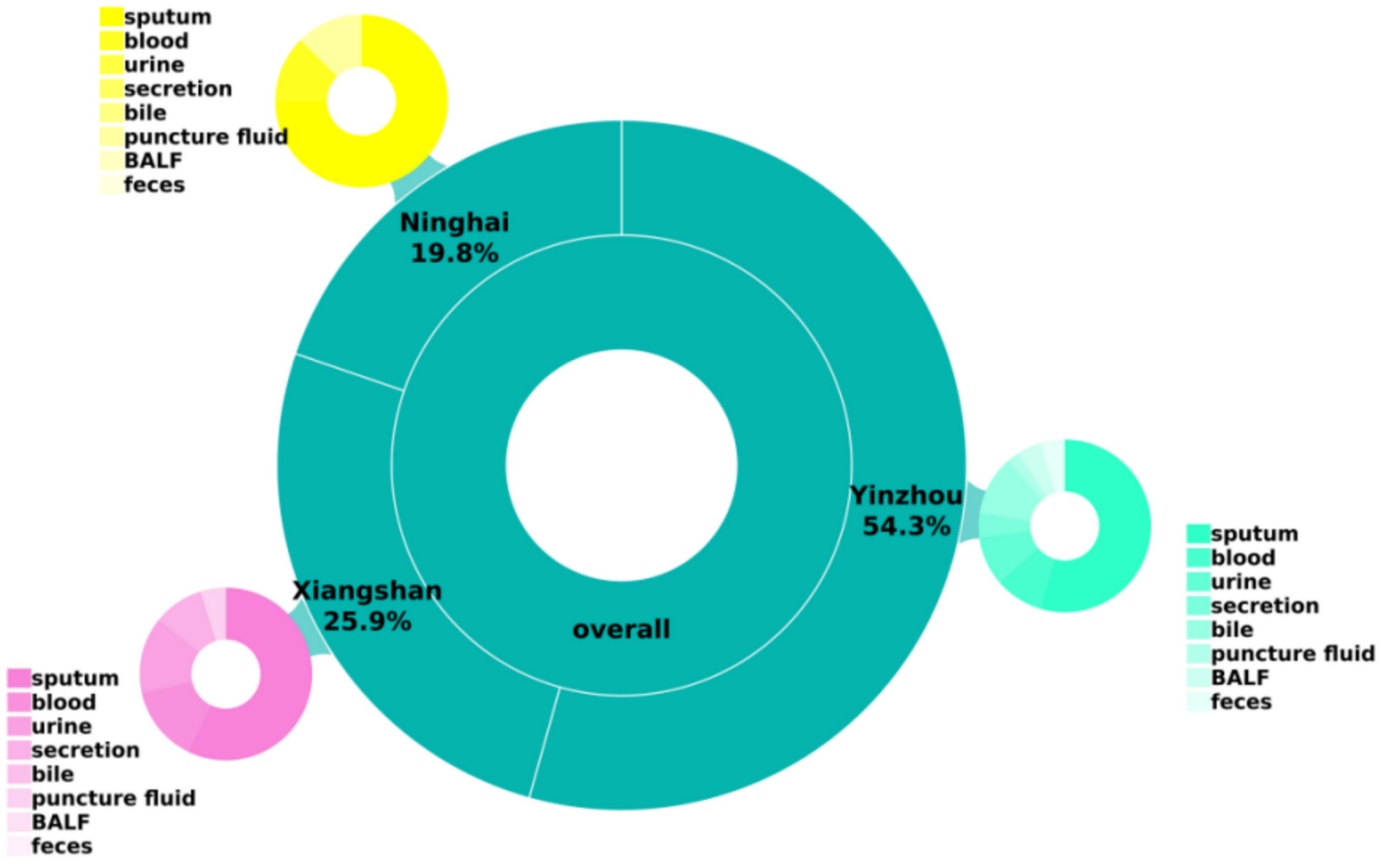
Basic clinical data of the strains.

A total of 81 carbapenem-resistant *Klebsiella pneumoniae* (CR-KP) isolates were collected in this study from multiple centers in the Ningbo region. The majority of specimens were derived from sputum, followed by blood, urine, and other body fluid samples, as shown in Table 2 and Figure 1.

**Table 2:** Clinical sources of the strains.

Sample type	Source		
	Yinzhou	Xiangshan	Ninghai
Sputum	24	12	12
Blood	4	3	2
Urine	4	3	0
Secretion	2	2	0
Bile	5	0	0
Puncture fluid	1	1	2
Bronchoalveolar lavage fluid (BALF)	2	0	0
Feces	2	0	0

### 3.2 MLST typing results of carbapenem-resistant *Klebsiella pneumoniae*

**Figure 2:**
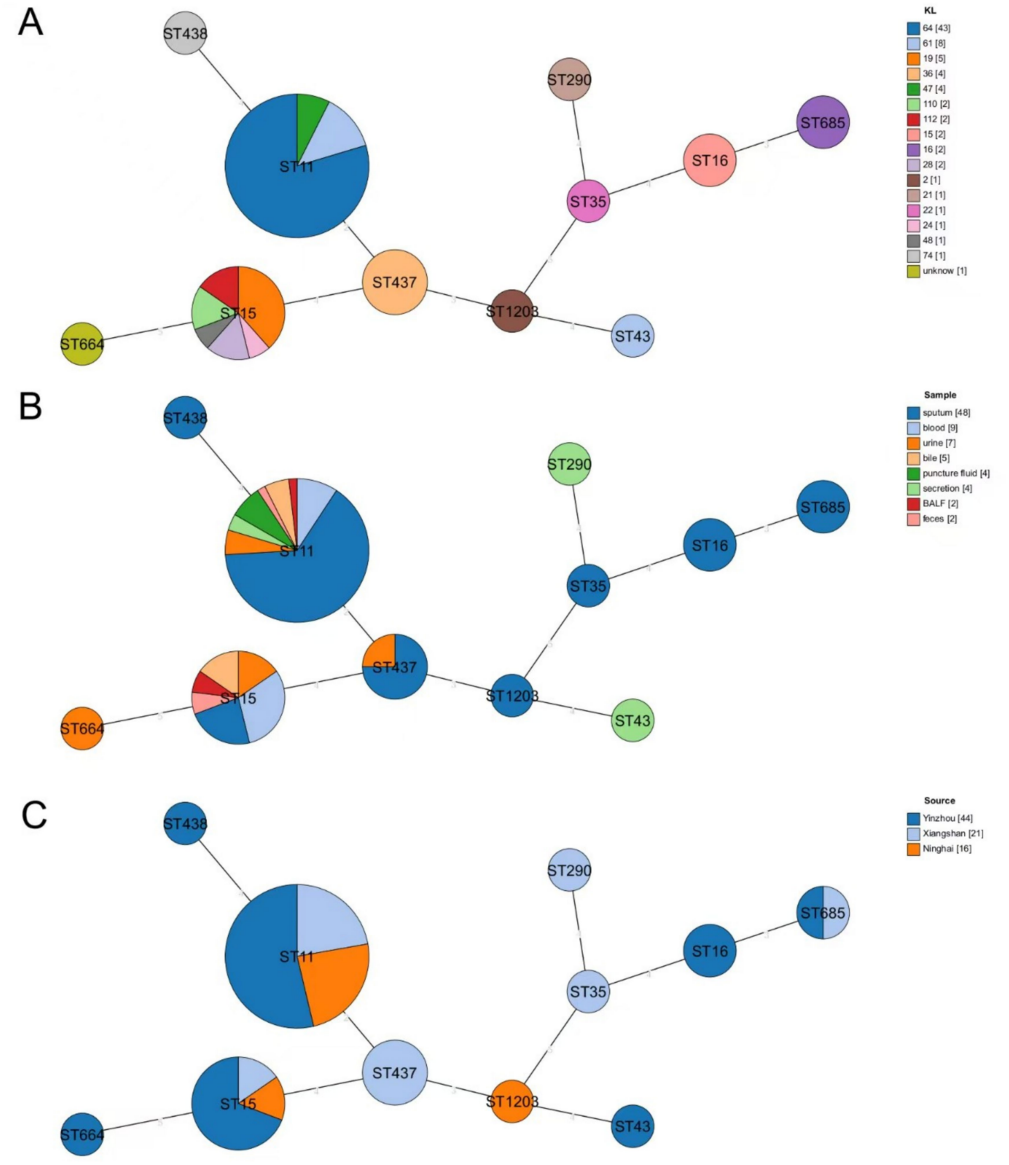
Minimum spanning tree based on MLST. **(A)** Distribution of STs of CR-KP in different KL isolates, with different colors representing different KL types. **(B)** Distribution of STs of CR-KP in isolates from different sample types, with different colors representing different sample types. **(C)** Distribution of STs of CR-KP in isolates from different collection sites, with different colors indicating different collection sites. Circles represent ST types, and the size of the circles indicates the number of strains in each ST.

Among the 81 CR-KP strains analyzed, 11 distinct sequence types (STs) were identified. As illustrated in Figure 2A, ST11 was the predominant clone, accounting for 66.67% (54/81) of isolates and distributed across 3 KL types, 3 isolation sites, and 8 sample types. The second most prevalent ST, ST15, represented 16.05% (13/81) of the strains. KL typing (Figure 2A) revealed KL64 as the dominant type (53.09%, 43/81), exclusively associated with ST11 and detected in 3 isolation sites and all 8 sample types. Regarding sample sources (Figure 2B), sputum constituted the majority (59.26%, 48/81) and encompassed most of STs, with ST11 and ST15 being the most frequently observed across all sample categories. Geographically (Figure 2C), Ninghai exhibited limited ST diversity, with only three STs detected (ST11, ST15, and ST1203), whereas Xiangshan and Yinzhou displayed higher strain heterogeneity, harboring 6 and 7 distinct STs, respectively.

### 3.3 Detection results of virulence-associated genes

Among the 81 CRKP strains, all carried multiple virulence factors, but there was a significant difference in the carriage rate of virulence genes between the ST11 type and non-ST11 type groups. The virulence genes detected in ST11 CR-KP isolates included: (1) Capsule-enhancing genes *rmpA* (3.70%, 2/54), *rmpA2* (77.78%, 42/54); (2) Siderophore-related genes included *fyuA* (98.15%, 53/54)*, irp1* (96.30%, 52/54)*, irp2* (98.15%, 53/54)*, iucA* (77.78%, 42/54), *iucB* (77.78%, 42/54)*, iucC* (77.78%, 42/54)*, iucD* (77.78%, 42/54)*, iutA* (77.78%, 42/54)*, ybtS* (94.44%, 51/54); (3) Fimbriae synthesis and adhesion-associated genes *mrkABCD* were all present in 100% of the isolates (54/54).

Among the non-ST11 CR-KP isolates, the detected virulence genes included: (1) Capsule-enhancing gene *rmpA2* (7.41%, 2/27); (2) Siderophore-related genes: *fyuA* (62.96%, 17/27)*, irp1* (25.93%, 7/27)*, irp2* (55.56%, 15/27)*, iucA* (37.04%, 10/27)*, iucB* (37.04%, 10/27), *iucC* (37.04%, 10/27), *iucD* (37.04%, 10/27), *iutA* (37.04%, 10/27), *ybtS* (62.96%, 17/27); (3) Fimbriae synthesis and adhesion-associated genes: *mrkA* (77.78%, 21/27)*, mrkB* (92.59%, 25/27)*, mrkC* (96.30%, 26/27)*, mrkD* (85.19%, 23/27), *allS* (7.41%, 2/27) and *allR* (7.41%, 2/27).

**Figure 3:**
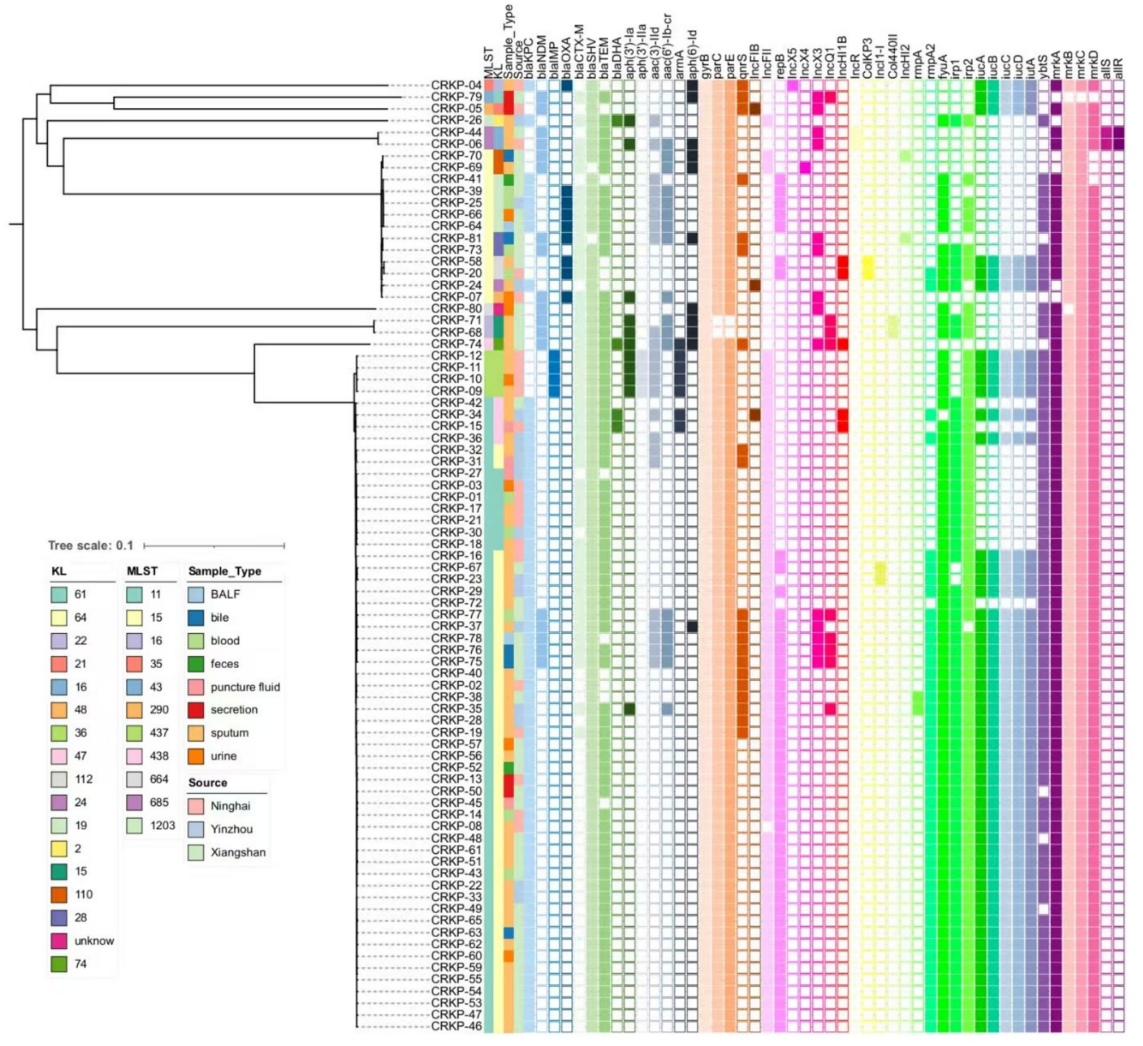
Phylogenetic of virulence-associated genes, antibiotic resistance, and plasmids. A core genome SNP-based phylogenetic analysis was performed to assess genetic relationships among 81 *Klebsiella pneumoniae* clinical isolates. The resulting phylogenetic tree illustrates evolutionary divergence through branch lengths, with terminal clades representing individual strains. An adjacent annotation panel employs color-coded blocks (arranged left-to-right) to visualize the distribution of: (1) antimicrobial resistance determinants, (2) plasmid replicon types, and (3) virulence-associated genes. Color gradients correspond to specific genetic elements, while blank spaces indicate their absence in respective isolates.

In summary, among the 17 virulence genes screened, ST11 CR-KP isolates exhibited significantly higher carriage rates of *rmpA2*; siderophore-associated genes *fyuA, irp1, irp2, iucA, iucB, iucC, iucD, iutA, ybtS*; and fimbriae/adhesion genes *mrkA* and *mrkD* compared to non-ST11 isolates, wherein all variations proved statistically robust (*p* < 0.05), as shown in Table 3 and Figure 3.

**Table 3:** The percentage of virulence genes carried by CR-KP.

Virulence genes	ST11 CR-KP (*n* = 54)		Non-ST11 CR-KP (*n* = 27)		χ^2^ value	*p* value
	Strains (n)	Percentage (%)	Strains (n)	Percentage (%)		
*rmpA*	2	3.70	0	0.00	–	0.550
*rmpA2*	42	77.78	2	7.41	35.923	<0.001
*fyuA*	53	98.15	17	62.96	16.108	<0.001
*irp1*	52	96.30	7	25.93	45.055	<0.001
*irp2*	53	98.15	15	55.56	21.178	<0.001
*iucA*	42	77.78	10	37.04	12.999	<0.001
*iucB*	42	77.78	10	37.04	12.999	<0.001
*iucC*	42	77.78	10	37.04	12.999	<0.001
*iucD*	42	77.78	10	37.04	12.999	<0.001
*iutA*	42	77.78	10	37.04	12.999	<0.001
*ybtS*	51	94.44	17	62.96	11.007	<0.001
*mrkA*	54	100.00	21	77.78	9.923	0.002
*mrkB*	54	100.00	25	92.59	–	0.108
*mrkC*	54	100.00	26	96.30	–	0.333
*mrkD*	54	100.00	23	85.19	5.556	0.018
*allS*	0	0.00	2	7.41	–	0.108
*allR*	0	0.00	2	7.41	–	0.108

### 3.4 Antimicrobial susceptibility of carbapenem-resistant *Klebsiella pneumoniae*

The drug susceptibility testing revealed that all 81 CRKP strains exhibited extensive resistance to commonly used clinical antimicrobial agents. Notably, the prevalence of multi drug-resistant (MDR) strains was particularly high at 91.36% (74/81), though no extensively drug-resistant (XDR) or pan drug-resistant (PDR) strains were identified. The resistance profile demonstrated consistently high resistance rates to cephalosporins, aminoglycosides, and quinolones. However, promising susceptibility was observed for three agents: aztreonam/avibactam, polymyxin B, and tigecycline, which remained highly effective against these resistant strains (Table 4).

**Table 4:** Antimicrobial susceptibility of CR-KP.

Antimicrobials	ST11 *C*R-KP (*n* = 54)				Non-ST11 CR-KP (*n* = 27)				χ^2^ value	*P* value
	Resistant strains (n)	%	MIC50	MIC90	Resistant strains (n)	%	MIC50	MIC90		
CTT	54	100.00	256	≥256	20	74.07	128	≥256	12.216	<0.001
CRO	54	100.00	≥128	≥128	27	100.00	≥128	≥128	–	–
FEP	54	100.00	≥128	≥128	25	92.59	128	≥128	–	0.108
TZP	54	100.00	≥256/4	≥256/4	24	88.89	≥256/4	≥256/4	3.505	0.061
IPM	54	100.00	32	64	27	100.00	32	128	–	–
GEN	1	1.85	1	2	11	40.74	2	≥256	18.599	<0.001
LVX	54	100	≥16	≥16	24	88.89	16	≥16	3.505	0.061
CIP	54	100	≥16	≥16	24	88.89	≥16	≥16	3.505	0.061
AZA	0	0.00	0.5/4	1/4	0	0.00	0.125/4	0.5/4	–	–
CZA	5	9.26	3	12	17	62.96	256	256	26.241	<0.001
POL	2	3.70	0.5	1	0	0.00	0.5	1	–	0.550
TGC	0	0.00	0.5	1	0	0.00	0.125	1	–	–

S, susceptible; I, intermediate; R, resistance; MIC, minimum inhibitory concentration; MIC50/MIC90, MIC50 and MIC90 can inhibit 50% or 90% of the tested bacteria.

ST11-type CR-KP isolates exhibited 100% resistance to cefotetan (CTT), ceftriaxone (CRO), cefepime (FEP), piperacillin/tazobactam (TZP), imipenem (IPM), levofloxacin (LVX), and ciprofloxacin (CIP). However, the resistance rates to gentamicin (GEN), aztreonam/avibactam (AZA), polymyxin B (POL), ceftazidime/avibactam (CZA), and tigecycline (TGC) were all below 10%. Notably, all ST11 CR-KP isolates tested in this study were 100% susceptible to aztreonam/avibactam (AZA) and tigecycline (TGC), suggesting that for ST11 CR-KP infections in Ningbo, empirical treatment with AZA or TGC may be considered as a frontline clinical option.

Non-ST11 CR-KP isolates showed 100% resistance to ceftriaxone (CRO) and imipenem (IPM), and approximately 75% resistance rates to cefotetan (CTT), piperacillin/tazobactam (TZP), cefepime (FEP), levofloxacin (LVX), ciprofloxacin (CIP) and approximately 62.96% resistance rates to ceftazidime/avibactam (CZA). However, these isolates were 100% susceptible to aztreonam/avibactam (AZA), polymyxin B (POL) and tigecycline (TGC).

In the antimicrobial susceptibility testing, except for ceftazidime/avibactam, gentamicin and imipenem, the MIC50 and MIC90 values of cefotetan, tigecycline, and aztreonam/avibactam in ST11-type CR-KP isolates were all higher than those in non-ST11 CR-KP isolates. Specifically, the MIC50 for cefotetan in ST11 CR-KP was 256 μg/mL, which was twice that of non-ST11 CR-KP (128 μg/mL); Tigecycline showed an MIC50 of 0.5 μg/mL, which is quadruple those in non-ST11 CR-KP (MIC50: 0.125 μg/mL). Moreover, aztreonam/avibactam exhibited an MIC50 of 0.5/4 μg/mL in ST11 CR-KP, four times higher than that of non-ST11 CR-KP (0.125/4 μg/mL). Additionally, except for gentamicin, the resistance rates to cefotetan, levofloxacin and ciprofloxacin were significantly higher in ST11 CR-KP compared to non-ST11 CR-KP, indicating a stronger resistance profile in ST11 CR-KP. These findings are consistent with the study by Pu et al. (2023b).

### 3.5 Comparison of resistance phenotypes and genotypes in carbapenem-resistant *Klebsiella pneumoniae*

Among the 54 ST11-type CR-KP isolates, the concordance rate between resistance phenotypes and genotypes across seven antimicrobial agents reached 97.35% (368/378). Notably, among the isolates carrying aminoglycoside resistance genes, nine strains (CR-KP15, CR-KP31, CR-KP32, CR-KP36, CR-KP37, CR-KP75, CR-KP76, CR-KP77, CR-KP78) exhibited phenotypic susceptibility to gentamicin, and one strain (CR-KP34) displayed intermediate susceptibility to gentamicin.

Comparison of the resistance phenotypes and genotypes of 27 non-ST11 CR-KP strains against seven antimicrobial agents revealed a relatively low overall concordance rate of only 83.60% (158/189). Discrepancies between phenotypic and genotypic resistance were primarily manifested in the following aspects: among strains carrying β-lactamase resistance genes, four were susceptible to cefotetan (CR-KP20, CR-KP25, CR-KP39, CR-KP58), and three exhibited intermediate susceptibility to cefotetan (CR-KP06, CR-KP24, CR-KP71); among these same β-lactamase gene-harboring strains, two demonstrated intermediate susceptibility to cefepime (CR-KP26, CR-KP39). Additionally, among strains harboring aminoglycoside resistance genes, 13 were susceptible to gentamicin (CR-KP04, CR-KP07, CR-KP25, CR-KP26, CR-KP39, CR-KP41, CR-KP64, CR-KP66, CR-KP69, CR-KP70, CR-KP79, CR-KP80, CR-KP81); among strains carrying quinolone resistance genes, two were susceptible to levofloxacin (CR-KP04, CR-KP80), one showed intermediate susceptibility to levofloxacin (CR-KP05), one was susceptible to ciprofloxacin (CR-KP04), two showed intermediate susceptibility to ciprofloxacin (CR-KP05, CR-KP80), Notably, three strains (CR-KP20, CR-KP44, CR-KP58) that did not carry any known aminoglycoside resistance genes were nonetheless resistant to gentamicin. We speculate that this phenomenon of genotype–phenotype discordance may be attributed to the complexity and heterogeneity of bacterial resistance mechanisms. Our current findings are insufficient to fully elucidate all resistance mechanisms, underscoring the urgent need for further in-depth investigations. Detailed antimicrobial susceptibility data for the isolates are provided in Supplementary Table S1.

Carbapenemase gene detection revealed that ST11 CR-KP strains predominantly harbored class A carbapenemase *bla_KPC-2_*, with no detection of *bla*_OXA_ or *bla*_IMP_. Notably, five isolates co-harbored the *bla_KPC-2_* and *bla_NDM-5_* resistance plasmids; In contrast, non-ST11 CR-KP strains were primarily associated with class B carbapenemases, among which *bla*_NDM_ accounted for 48.15% (13/27), *bla_IMP_* for 14.81% (4/27), and class D carbapenemase *bla*_OXA_ for 33.33% (9/27). Multiple carbapenemase resistance genes were frequently co-detected, including four isolates co-harboring *bla*_KPC-2+_*bla*_OXA-1_ (CR-KP25, CR-KP39, CR-KP64, CR-KP66); and single isolates carrying *bla*_KPC-3+_*bla*_OXA-10_ (CR-KP04), *bla*_NDM-1+_*bla*_OXA-1_ (CR-KP81), *bla*_NDM-5+_*bla*_OXA-1_ (CR-KP07). Detailed data are presented in Table 5 and Figure 3; full enzyme profile information is available in Supplementary Table S1.

**Table 5:** The percentage of antibiotic resistance genes in CR-KP.

Resistance genes	ST11 CR-KP (*n* = 54)		Non-ST11 CR-KP (*n* = 27)		χ^2^ value	*P* value
	Strains (n)	Percentage (%)	Strain (n)	Percentage (%)		
β-lactamase						
*bla*_CTX-M_	23	42.59	22	81.48	11.025	<0.001
*bla*_SHV_	54	100.00	25	92.59	–	0.108
*bla*_TEM_	48	88.89	21	77.78	0.990	0.320
*bla*_DHA_	2	3.70	2	7.41	0.033	0.856
Carbapenemases						
*bla*_KPC_	54	100.00	8	29.63	49.645	<0.001
*bla*_NDM_	5	9.26	13	48.15	15.750	<0.001
*bla*_IMP_	0	0.00	4	14.81	5.556	0.018
*bla*_OXA_	0	0.00	9	33.33	17.016	<0.001
Quinolone resistance genes						
*gyrB*	54	100.00	27	100.00	–	–
*parC*	54	100.00	25	92.59	–	0.108
*parE*	54	100.00	25	92.59	–	0.108
*qnrS*	13	24.07	7	25.93	0.033	0.855
Aminoglycoside resistance genes						
*aph(3′)-Ia*	1	1.85	10	37.04	16.108	<0.001
*aph(3′)-IIa*	0	0.00	4	14.81	5.556	0.018
*aac(3)-IId*	9	16.67	14	51.85	10.960	<0.001
*aac(6′)-Ib-cr*	6	11.11	12	44.44	11.571	<0.001
*armA*	2	3.70	5	18.52	3.303	0.069
*aph(6)-Id*	1	1.85	10	37.04	16.108	<0.001
Tetracycline resistance genes‌						
*tet(A)*	31	57.41	15	55.56	0.025	0.874
*tmexD1*	0	0.00	1	3.70	–	0.333
*TOprJ1*	0	0.00	1	3.70	–	0.333

**Figure 4:**
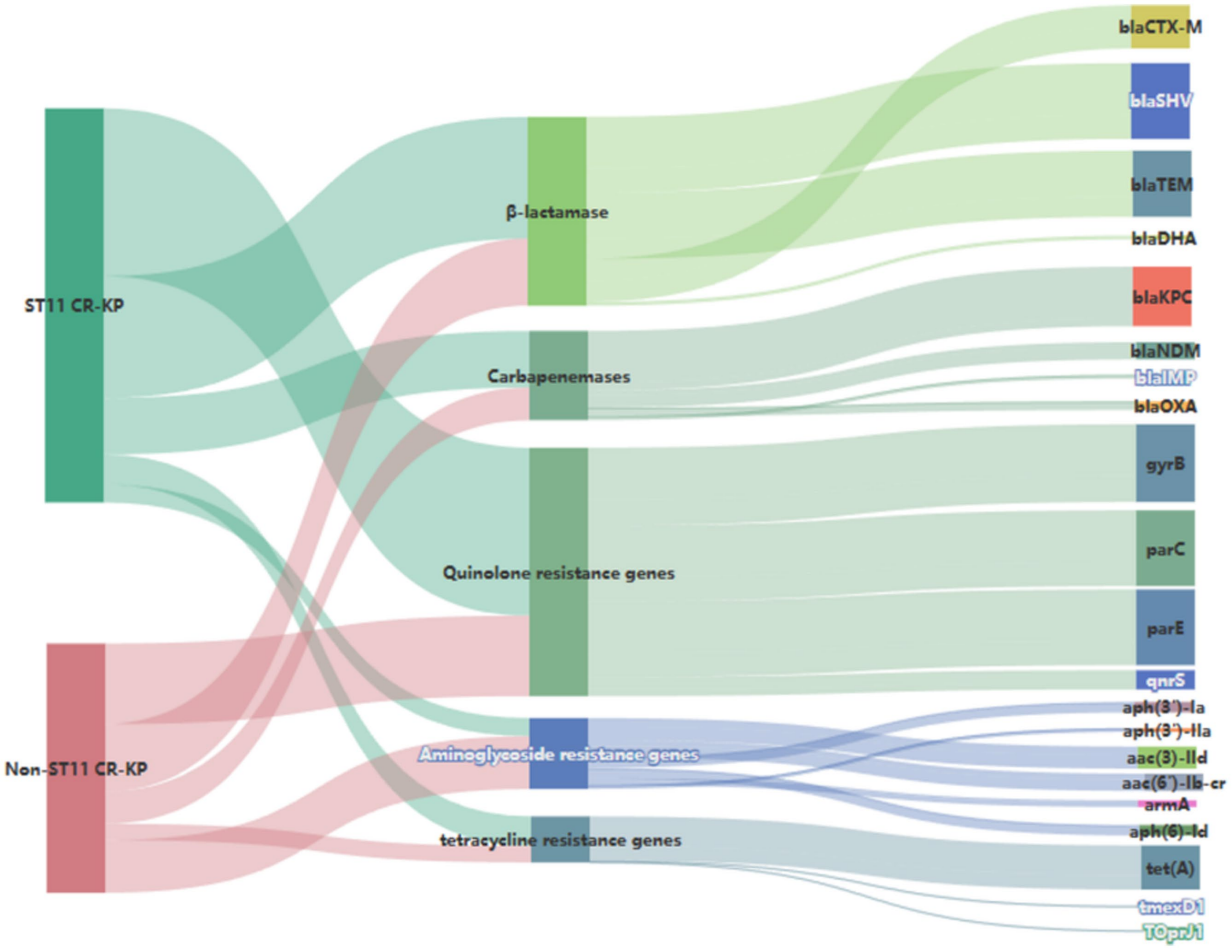
Sankey diagram of antibiotic resistance genes in CR-KP. A Sankey diagram also known as a Sankey energy flow chart, is used here. The left nodes represent non-ST11 and ST11 types of CR-KP, while the right nodes represent five antimicrobial classes (including 22 kinds of antibiotic resistance genes) the width of the branches correlates with number of resistance genes.

Genetic investigation of β-lactamase resistance markers revealed that ST11 CR-KP isolates exhibited markedly elevated frequencies of *bla*_SHV_ and *bla*_TEM_ compared to non-ST11 variants, whereas *bla*_CTX-M_ demonstrated increased prevalence in non-ST11 strains. Concerning quinolone resistance genetic markers, the prevalence of *gyrB, parC, parE*, or *qnrS* revealed no statistically meaningful disparities between the comparative groups (*P* > 0.05). Regarding aminoglycoside resistance genes, non-ST11 CR-KP strains exhibited significantly higher carriage rates of *aph (3′)-Ia, aph(3′)-IIa, aac(3)-IId, aac(6′)-Ib-cr*, and *aph(6)-Id* compared to ST11 strains (*p* < 0.05), which was consistent with the higher resistance phenotype to gentamicin observed in non-ST11 CR-KP strains in the antimicrobial susceptibility test. These findings are presented in Table 5, Figures 3, 4.

### 3.6 Plasmid profiles of carbapenem-resistant *Klebsiella pneumoniae*

**Figure 5:**
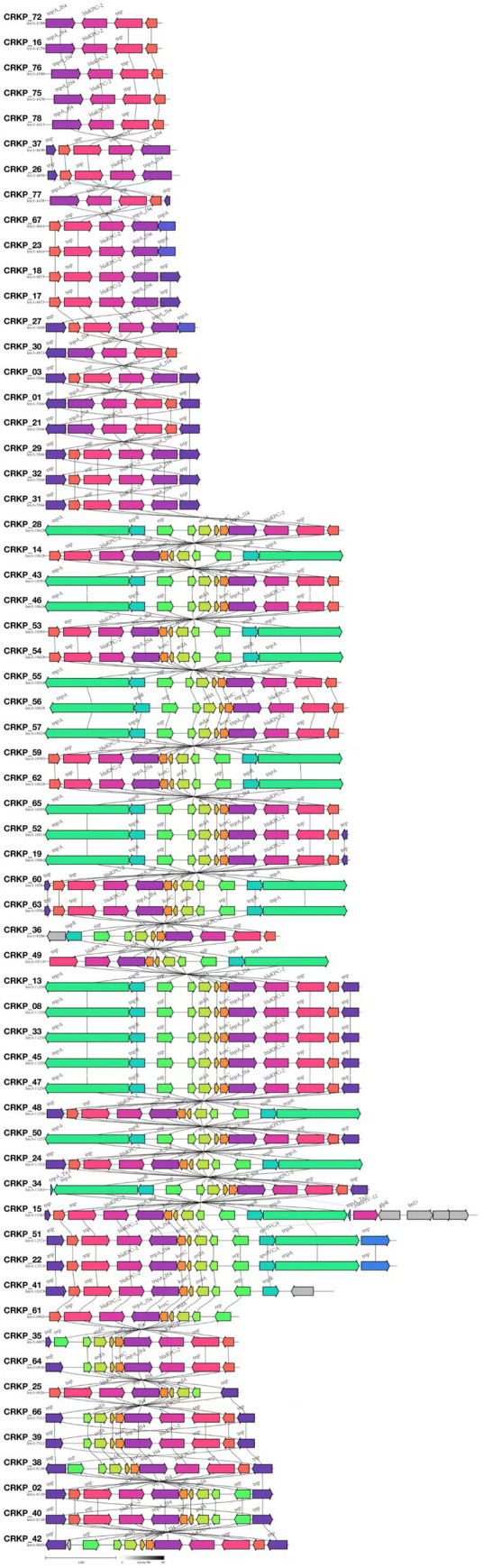
Comparative genomic architecture of *bla*_KPC-2_ loci in CR-KP. In the annotated genetic map, colored arrows represent predicted genes or open reading frames (ORFs), with arrow directionality indicating transcriptional orientation and color gradients distinguishing functional gene categories. All 61 isolates carrying the *bla*_KPC-2_ gene were analyzed using Illumina short-read sequencing due to budget constraints.

Among the 81 CR-KP isolates analyzed in this study, the major plasmid types detected included IncFII, repB, IncX3, and IncQ1. In ST11 CR-KP strains, IncFII (98.15%, 53/54) and repB (72.22%, 39/54) were the predominant plasmid types. In contrast, non-ST11 CR-KP strains carried a more diverse range of plasmid types, including IncX3 (33.33%, 9/27), repB (25.93%, 7/27), IncFII (25.93%, 7/27), IncQ1 (14.81%, 4/27), and ColKP3 and Col440II (7.41%, 2/27 each). The greater plasmid diversity observed in non-ST11 CR-KP strains was associated with the co-occurrence of multiple carbapenem resistance genes. Plasmid carriage results for both groups are shown in Figure 3, and detailed plasmid typing data are provided in Supplementary Data S1. Furthermore, this study conducted a colinearity analysis of the *bla*_KPC-2_ resistance plasmid gene (Figure 5). This figure comprehensively displays the sequences containing the *bla*_KPC-2_ gene from 61 libraries, with each sequence clearly harboring the critical antibiotic resistance gene *bla*_KPC-2_. The figure shows the genetic organization of the *bla*_KPC-2_ gene and its adjacent elements, including common accompanying genes such as transposase genes. The repeated occurrence of these genes underscores the high mobility and complex transmission mechanisms of the *bla*_KPC-2_ gene. The presence of scaffolds sharing the *bla*_KPC-2_ gene, along with potential auxiliary genes upstream and downstream, collectively reveal the complexity of *bla*_KPC-2_ gene transmission and replication in *Klebsiella pneumoniae*.

### 3.7 Analysis of the phylogenetic tree results based on wgSNP

We constructed a phylogenetic tree using the GTR model based on whole-genome SNPs (wgSNPs) of 81 *Klebsiella pneumoniae* genomes. As shown in Figure 3, the wgSNP-based phylogenetic tree clearly illustrates genetic distances among strains, where those with the same MLST typing (i.e., branches of the same color) tend to cluster in the evolutionary tree. For example, dominant ST11 strains (e.g., CRKP-46, −37, −16) mostly gather in a major lower branch, while green-colored ST437 strains (e.g., CRKP-09, −11, −12) above ST11 form a distinct branch. Another dominant ST15 cluster comprises a large branch (e.g., CRKP-70, -69, -64), whereas smaller ST types (ST685, ST43, ST16, etc.) form smaller branches. These results confirm that while different MLST types generally cluster in the wgSNP tree—thereby verifying MLST’s effectiveness in distinguishing major genetic lineages—wgSNP analysis reveals substantial genetic diversity within the same MLST type. For instance, ST15 strains, despite sharing the same MLST classification, exhibit obvious branching and genetic distances in the wgSNP tree, indicating that traditional MLST—relying on only a few housekeeping genes—lacks the resolution to distinguish such subtle genetic variations.

### 3.8 String test

A string test was conducted on 81 CRKP isolates, which revealed that only five strains exhibited positive string formation on blood agar plates (Oxoid, Hampshire, UK), indicative of the hypermucoviscous phenotype.

## 4 Discussion

By analyzing the molecular characteristics and antimicrobial susceptibility profiles of 81 CR-KP isolates from Ningbo, this study found that ST11-KL64 was the predominant CR-KP type in the region. This epidemiological pattern aligns with the findings of Wang et al. (2023) and Wang et al. (2024), who reported the dominance of ST11-KL64 strains in both China and globally. In contrast, non-ST11 CR-KP strains were mainly of the ST15-KL19 type, differing from the findings of Feng et al. (2023), who reported ST15 CR-KP predominantly associated with KL112. These results suggest potential regional variation in the evolution of capsular gene types in *Klebsiella pneumoniae*.

Analysis of virulence genes revealed that the ST11-type CR-KP exhibited significantly higher carriage rates (*p* < 0.05) of the capsule-promoting gene *rmpA2*; siderophore-related genes *fyuA, irp1, irp2, iucA, iucB, iucC, iucD, iutA, ybtS*; and fimbriae assembly and adhesion-related genes *mrkA, mrkD*, compared to non-ST11 types. Previous studies have confirmed that the *rmpA2* gene enhances bacterial survival in the host by regulating the synthesis of capsular polysaccharide (CPS) (Kong et al., 2021; Huang et al., 2024). Siderophore systems (such as *fyuA, iutA, ybtS*) promote biofilm formation by chelating iron from the host environment (Zhu et al., 2021; Yu et al., 2024). The *mrkA* gene, associated with fimbrial assembly and adhesion, encodes the pilin subunit of type III fimbriae and plays a critical role in fimbrial structural formation. The *mrkD* gene encodes an adhesin that facilitates bacterial attachment to the extracellular matrix (Nazari et al., 2024). The presence of these genes enhances the colonization capability of ST11-type CR-KP in the host, thereby increasing the risk of infection. These findings are consistent with the study by Li L. et al. (2025), suggesting that ST11-type CR-KP possesses greater pathogenic potential.

In addition to virulence characteristics, we also analyzed the distribution of resistance genes: ST11-type CR-KP predominantly harbored the class A enzyme gene *bla*_KPC-2_, with a significantly higher carriage rate than that of non-ST11-type CR-KP (*p* < 0.05), aligning with the findings of Pu et al. (2023b), who also reported that ST11-type CR-KP primarily carries *bla*_KPC-2_. Among the ST11-type CR-KP isolates, five strains were found to co-harbor two carbapenemase genes, *bla*_KPC-2+_*bla*_NDM-5_, which is consistent with the findings of Li J. et al. (2025), who reported an increasing global prevalence of *bla*_KPC_ + *bla*_NDM_ producing CR-KP infections since 2020. Additionally, among the non-ST11-type CR-KP isolates, a total of seven strains carried two or more types of carbapenemase genes. These included four strains with *bla*_KPC-2+_*bla*_OXA-1_, and one strain each with *bla*_KPC-3+_*bla*_OXA-10_, *bla*_NDM-1+_*bla*_OXA-1_ and *bla*_NDM-5+_*bla*_OXA-1_. To date, both domestic and international reports have documented CR-KP strains co-producing two (Guo et al., 2023; Zhou et al., 2024; Mapunda et al., 2025; Phan et al., 2025) or multiple (Mataseje et al., 2022; Ghanbarinasab et al., 2023; Montenegro et al., 2023) carbapenemases, with common combinations including *bla*_NDM+_*bla*_OXA-48_, and *bla*_KPC-2+_*bla*_NDM-5_. Guo et al. (2023) analyzing 832 CR-KP strains carrying dual carbapenemase genes from the NCBI database, found that in China, co-production of *bla*_KPC_, and *bla*_NDM_ was predominant, which aligns with our findings. In contrast, strains from the United States and Thailand more commonly co-harbored *bla*_NDM_ and *bla*_OXA-48_, indicating regional differences in the genetic distribution of epidemic clones. Furthermore, among the 81 CR-KP isolates, in addition to carbapenemase genes such as *bla*_KPC-2_, *bla*_NDM_, *bla*_IMP_, *bla*_OXA_, multiple ESBL genes were also detected, including *bla*_CTX-M_, *bla*_SHV_, and *bla*_TEM_. The observed data align with the research conducted by Yin et al. (2022), which demonstrated that within *bla*_KPC-2_ producing *K. pneumoniae* strains, the predominant ESBL resistance genes included *bla*_CTX-M-14/15_, *bla*_SHV-11/12_ and *bla*_TEM-1_. The co-expression of carbapenemase and ESBL resistance genes significantly enhances the antimicrobial resistance of clinical isolates.

Moreover, the dissemination of resistance genes is closely associated with the diversity of plasmid types. ST11-type CR-KP primarily relies on IncFII and RepB plasmids for transmission, in alignment with the investigation by Liu et al. (2023), who demonstrated that *bla*_KPC-2_ dissemination is mediated by IncFII plasmids. In addition, several studies (Luo et al., 2024; Zhang F. et al., 2024) have found that RepB-type plasmids in CR-KP commonly co-carry and disseminate both resistance and virulence genes, thereby enhancing the pathogenicity and drug resistance of CR-KP. In contrast, non-ST11-type CR-KP tends to rely on broad-host-range plasmids, such as IncX3, and ColKP3, to facilitate horizontal transfer of resistance genes. Guo et al. (2022) reported that *bla*_NDM_ is typically located on IncX3 plasmids, which are considered the primary vectors for the spread of *bla*_NDM_. In the present study, all CR-KP strains carrying IncX3 plasmids also harbored *bla*_NDM-5_, which aligns with their findings. Furthermore, two strains in this study carrying ColKP3 plasmids were found to harbor the *bla*_OXA-232_ gene, parallel to the findings presented by Chukamnerd et al. (2022), which indicated that *bla*_OXA-232_ is typically located on ColKP3 plasmids. ColKP3 is a non-conjugative plasmid, but it can be mobilized in the presence of a co-residing conjugative plasmid. This highlights the need to closely monitor the risk of co-transfer involving non-conjugative plasmids.

Investigations of alternative resistance genetic markers revealed substantial differences in the distribution frequencies of aminoglycoside resistance genes across the compared CR-KP groups, the carriage rates of aminoglycoside resistance genes *aph (3′)-Ia, aph (3′)-IIa, aac (3)-IId, aac (6′)-Ib-cr, armA, aph (6)-Id* were higher in non-ST11-type CR-KP than in ST11-type CR-KP, which was consistent with the antimicrobial susceptibility phenotypes, non-ST11-type CR-KP demonstrated a significantly higher resistance rate to gentamicin than the ST11-type. Furthermore, in this study, although no tigecycline-resistant strains were detected, data from Table 5 and Figure 4 indicate that the carriage rate of the tetracycline resistance gene *tet(A)* in the ST11 group CRKP was 57.41% (31/54), while in the non-ST11 group CR-KP it was 55.56% (15/27) and the carriage rates of the *tmexD1* and *TOprJ1* genes were both 3.70% (1/27). It is noteworthy that previous studies have confirmed the prevalence of plasmid-borne *tet (A)* gene in clinical CR-KP, with *tet(A)* gene mutations or overexpression considered a potential driving factor for tigecycline resistance (Su et al., 2024). Further studies have shown that increases in tigecycline minimum inhibitory concentration (MIC) are closely related to the expression levels of *tet(A)* mutants (Xu et al., 2021). These findings suggest that although a tigecycline-resistant phenotype was not observed in this study, the high carriage rate of the *tet(A)* gene and its association with resistance mechanisms imply that this gene may pose a potential threat to the development of tigecycline resistance.

Antimicrobial susceptibility testing revealed that among 81 strains from the Ningbo area included in this study, 74 were identified as multi drug-resistant (MDR), accounting for 91.36% (74/81). Notably, no extensively drug-resistant (XDR) or pan drug-resistant (PDR) strains were detected. Specifically, the proportion of MDR strains among ST11 CR-KP was 100% (54/54), while that among non-ST11 CR-KP was 74.07% (20/27). Although the susceptibility rate of CR-KP to aztreonam/avibactam remains high in Ningbo, it is worth noting that this agent was only approved for marketing by the European Union in April 2024 and has not yet been authorized for clinical use in China. Therefore, accelerating the introduction of this antimicrobial agent is of great significance.

This study also found that both ST11-type and non-ST11-type CR-KP exhibited 100% susceptibility to tigecycline, suggesting that empirical use of tigecycline may be considered in critically ill patients with suspected CR-KP infections in Ningbo. Once routine antimicrobial susceptibility results are available, step-down therapy can be adjusted accordingly. However, our clinical screening has revealed the emergence of two polymyxin B resistant CR-KP strains in the Ningbo region. Most studies have shown that the resistance mechanism of colistin is extremely complex, mainly involving modifications to the lipid a component of lipopolysaccharide (LPS). These modification processes are typically driven by the upregulation of two-component regulatory systems (such as PmrAB and PhoPQ), which are achieved by adding cationic phosphoethanolamine (pEtN) or 4-amino-L-arabinose. In addition, the acquisition of plasmid-mediated mobile colistin resistance (*mcr*-1) genes (Mousavi et al., 2025; Zhang et al., 2025), along with various alterations in the *mgrB* gene (including: inactivation caused by an insertion sequences and nonsense point mutations) (da Silva et al., 2020), these all represent important resistance pathways. In the follow-up, we plan to expand the sample collection range to obtain more colistin-resistant strains, so as to carry out a more systematic and in-depth exploration of their resistance mechanisms. Although tigecycline and polymyxin B still maintain high levels of activity against CR-KP in the Ningbo region, both belong to the category of restricted antibiotics (Lyu et al., 2020; Tian et al., 2021), necessitating judicious clinical application and rigorous regulatory oversight to mitigate potential resistance development and transmission. For example, Huang et al. (2022) reported that co-administration of tigecycline with polymyxin during treatment could help prevent the development of resistance to polymyxin. Therefore, the implementation of antimicrobial stewardship programs (ASP), combination therapy strategies (Park et al., 2024; Park et al., 2025), and resistance surveillance can effectively manage the use of these critical antibiotics and help prevent the emergence of clinically resistant bacterial strains (Huang et al., 2022).

## 5 Conclusion

In summary, the predominant clonal group of CR-KP in the Ningbo region is the ST11-KL64 type, which exhibits a dual characteristic of “resistance and virulence,” presenting a critical challenge to hospital infection management and therapeutic effectiveness. The overall concordance between genotypic and phenotypic resistance patterns among the two CR-KP groups was high—97.35% for ST11-type and 83.60% for non-ST11-type—indicating that discrepancies between resistance gene profiles and antimicrobial susceptibility phenotypes are relatively uncommon in Ningbo. This highlights the potential of rapid enzymatic detection platforms, such as the GeneXpert system developed by Cepheid, to be effectively applied in the rapid microbiological diagnostics in this region. Such tools can support clinicians in making more scientific and rational antimicrobial choices early in the course of infection, thereby improving therapeutic outcomes. Notably, among the CR-KP strains identified with a “genotype–phenotype” discrepancy, 23 isolates carried aminoglycoside resistance genes but were phenotypically susceptible to gentamicin. Six isolates carried quinolone resistance genes but exhibited susceptibility or intermediate susceptibility to ciprofloxacin and levofloxacin in antimicrobial susceptibility testing. It is speculated that such “genotype–phenotype” discrepancies may be attributed to low expression levels of resistance genes. We plan to further investigate the underlying mechanisms, such as gene silencing or regulatory abnormalities, through quantitative real-time PCR (qRT-PCR) or whole-genome sequencing. In addition, we identified three CR-KP isolates that did not carry known aminoglycoside resistance genes but were phenotypically resistant to gentamicin, suggesting the potential existence of novel resistance mechanisms (Zhang et al., 2023). Interestingly, we alsofound that one strain without any carbapenemase showed a highly drug-resistant phenotype, suggesting it may be related to efflux pumps, porin loss, and regulatory mutations (Alenazy, 2022; Elías-López et al., 2024; Zhang L. et al., 2024) which warrants further in-depth investigation.

This study does have some limitations. Due to issues related to biosafety, standard operating procedures (SOPs), and other factors, it remains challenging to collect specimens from all districts in Ningbo. Currently, Ningbo is divided into six districts. In this study, we have made every effort to collect specimens from three districts. In the future, we will strengthen cooperation with other affiliated hospitals and strive to expand the sample size (e.g., ≥500 cases) and coverage (across all six districts).

## Data Availability

The nucleotide sequences of 81 CRKP isolates have been deposited in the GenBank database, with their respective accession numbers provided in Supplementary Table S1.
